# Determination of the Biological Activity and Structure Activity Relationships of Drugs Based on the Highly Cytotoxic Duocarmycins and CC-1065

**DOI:** 10.3390/toxins1020134

**Published:** 2009-12-02

**Authors:** Lutz F. Tietze, Birgit Krewer, J. Marian von Hof, Holm Frauendorf, Ingrid Schuberth

**Affiliations:** Institute of Organic and Biomolecular Chemistry, Georg-August-University Göttingen, Tammannstr. 2, 37077 Göttingen, Germany

**Keywords:** ADEPT, anticancer agents, cancer therapy, CC-1065, cytotoxicity, DNA alkylation, duocarmycins, electrospray mass spectrometry, HPLC, oligonucleotides, structure activity relationship

## Abstract

The natural antibiotics CC‑1065 and the duocarmycins are highly cytotoxic compounds which however are not suitable for cancer therapy due to their general toxicity. We have developed glycosidic prodrugs of *seco*-analogues of these antibiotics for a selective cancer therapy using conjugates of glycohydrolases and tumour-selective monoclonal antibodies for the liberation of the drugs from the prodrugs predominantly at the tumour site. For the determination of structure activity relationships of the different *seco*-drugs, experiments addressing their interaction with synthetic DNA were performed. Using electro­spray mass spectrometry and high performance liquid chromatography, the experiments revealed a correlation of the stability of these drugs with their cytotoxicity in cell culture investigations. Furthermore, it was shown that the drugs bind to AT-rich regions of double-stranded DNA and the more cytotoxic drugs induce DNA fragmentation at room temperature in several of the selected DNA double-strands. Finally, an explanation for the very high cytotoxicity of CC-1065, the duocarmycins and analogous drugs is given.

## 1. Introduction

**Figure 1:**
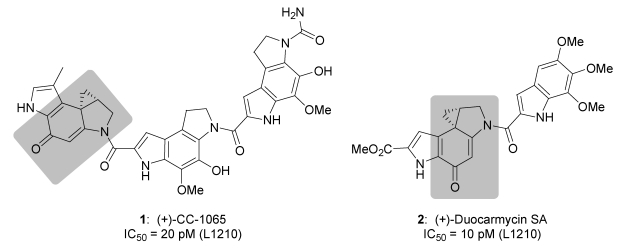
(+)-CC-1065 (**1**) and (+)-duocarmycin SA (**2**) with highlighted pharmacophoric units.

The natural antibiotics CC‑1065 (**1**, Figure 1) and the duocarmycins such as duocarmycin SA (**2**) are highly cytotoxic compounds and several derivatives of these natural products have already entered clinical trials [1,2,3,4]. Unfortunately, however, the general toxicity of these drugs is very high since their selectivity for tumour cells is rather low. The mode of action of the natural products as well as of related toxins is supposed to involve a sequence selective alkylation of cellular DNA in AT-rich regions that induces cell death [3,4,5].

**Figure 2:**
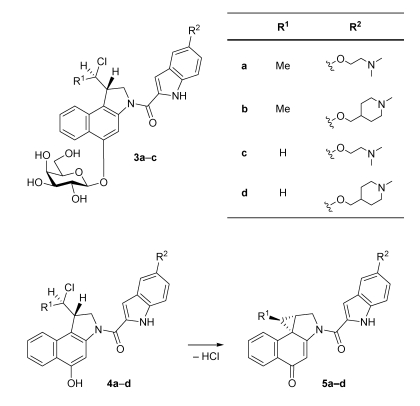
Glycosidic prodrugs **3a**-**c**, *seco*-drugs **4a**-**d** and cyclisation of the latter under loss of HCl to give the drugs **5a**-**d** as analogues of the natural products **1** and **2**.

To overcome the insufficient selectivity of these compounds, we have developed glycosidic prodrugs such as **3a**, **3b** and **3c** (Figure 2) [6,7,8,9]. The glycosidic bonds in **3a**-**c** can be cleaved selectively at the tumour site by using a suitable monoclonal-antibody-glycohydrolase-conjugate (here *β*-d-galactosidase) that removes the sugar moiety to give the corresponding *seco*-drugs **4a**-**c**. Due to the monoclonal-antibody, the conjugate binds to tumour-associated antigens, thus limiting the activation of the prodrugs to the tumour tissue [10,11]. The *seco-*drugs **4a**-**c** as well as **4d** cyclise rapidly with loss of HCl to give the corresponding drugs **5a**-**d** which contain a spirocyclopropyl-cyclo­hexa­dienone moiety similar to the one that can be found in the natural products **1**  and **2** (Figure 1). The alkylating moiety of **5c** and **5d** (R^1^ = H), which is formed *in situ*, is the 1,2,9,9a-tetra­hydro­cyclo­propa[1,2‑c]benz[1,2-e]indol-4-one (CBI) subunit developed by *Boger et al.* [12,13,14,15], whereas **5a** and **5b** (R^1^ = Me) bear the methylated CBI subunit developed in our group [6,7].

Whereas prodrugs **3a**, **3b** and **3c** showed an excellent selectivity in cell culture experiments with QIC_50_ (QIC_50_ = IC_50_ (prodrug)/IC_50_ (prodrug + enzyme)) values of 4800 (**3a**), 1100 (**3b**) and 3500 (**3c**) [6,7,8], compounds **3a** and **3b** exhibited a reduced stability when being incubated in cell culture media or phosphate buffer [16]. The obviously relatively easy replacement of the secondary chloro atom in **3a** and **3b** by a hydroxyl group, which renders the prodrugs inactive, resulted in a half life of approximately 14 h for these prodrugs. In contrast, **3c** showed less then 1% of hydrolysis after incubation for 24 h at 37 °C in cell culture medium [8,17]. 

Investigations of the reactivity of **4a** against synthetic models of potential cellular target molecules using electrospray ionisation Fourier transform ion cyclotron resonance mass spectrometry (ESI-FTICR MS) had shown a highly effective alkylation of double-stranded DNA by the drug **5a** formed *in situ* from **4a** [16,18]. In contrast, the reactivity of **5a** against single-stranded DNA, double-stranded RNA and the tripeptide glutathione was only low, suggesting that alkylation of double-stranded DNA is the predominant mode of action of this kind of drugs [19]. Since the DNA alkylation of analogue drugs did not correlate quantitatively with their relative cytotoxicity, we assumed that parameters like differences in the sequence selectivity [20,21] or in the stability of the compounds under physiological conditions [22,23] might modulate their cytotoxicity.

Here we describe investigations aiming at a better understanding of structure-activity relationships regarding this class of compounds. For these studies, the reactivity of the four *seco*-drugs **4a**-**d** against ten different DNA oligonucleotides was investigated using electrospray ionisation Fourier transform ion cyclotron resonance (ESI-FTICR) mass spectrometry and high performance liquid chromato­graphy (HPLC).

## 2. Results and Discussion

The cytotoxicity of the hydrochlorides of *seco*-drugs **4a**-**4d** against human bronchial carcinoma cells of line A549 (Table 1) was determined using a modified tumour colony forming ability test as reported previously [6,7,8,9,24].

**Table 1:** *In vitro* cytotoxicity of the hydrochlorides of *seco*-drugs **4a**-**d** against human bronchial carcinoma cells of line A549. Cells were exposed to various concentrations of the test substance for 24 h at 37 °C; after 12 days of subsequent incubation the clone formation was compared to an untreated control assay and the relative clone forming rate was determined. IC_50_: Concentration required for 50% growth inhibition of target cells.

*seco*-drug	IC_50_ (pm)
**4a**·HCl	750
**4b**·HCl	800
**4c**·HCl	26
**4d**·HCl	14

The *seco*-drugs **4c** and **4d** containing a hydrogen atom at the pharmacophoric unit (R^1^ = H) were found to show a much higher cytotoxicity than their analogues **4a** and **4b** containing a methyl group (R^1^ = Me) at the same position. This is presumably due to the fact that an attack of the cellular target, namely *N*3 of the nucleobase adenine, at the electrophilic cyclopropane moiety of the corresponding drugs **5a**-**d** resulting in a subsequent formation of the corresponding DNA adducts, is less hindered in the absence of the sterically more demanding methyl group. Additionally, the lower hydrolytic stability of the drugs **5a** and **5b** bearing a methyl group decreases their concentration in cell culture media [16,17], which would lower the cytotoxicity of these drugs determined in cell culture assays. The different side chains (R^2^) in **4a** and **4b** on the one hand and **4c** and **4d** on the other hand seem to have only a small influence on the drugs’ cytotoxicity since **4a** and **4b** as well as **4c** and **4d**, respectively, show cytotoxicities within the same order of magnitude. This is understandable because both side chains contain a tertiary amine and have approximately the same size. Denny *et al.* as well as Boger *et al.* have previously reported that substituents at C-5 of the DNA binding subunit, which are here represented by R^2^, have a pronounced effect on the rate and efficiency of the DNA alkylation and the resulting biological potency of CC‑1065 analogues [25,26,27,28], but the exact type of the substituent does not seem to be as important as its length. 

### 2.1. Investigations on the Reactivity and Sequence Selectivity of **4a-d** against DNA Oligomers

**Figure 3:**
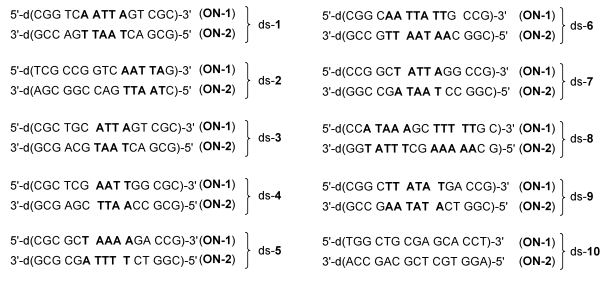
Base sequences of the ten double-stranded DNA oligonucleotides used in the present study. Double-stranded DNA oligonucleotides are denoted with the praefix “ds-“. Oligonucleotides with lower molecular mass are denoted with the abbreviation “**ON**-**1**” and oligonucleotides with higher molecular mass with the abbreviation “**ON**-**2**”. A: Adenine, G: Guanine, C: Cytosine, T: Thymine.

The reactivity and sequence selectivity of **4a**-**d** against ten different synthetic double-stranded DNA oligomers (ds-**1**-ds‑**10**) and against the synthetic single-stranded DNA oligo­nucleo­tide **ON**-**1** of ds‑**1** (Figure 3) was investigated using ESI-FTICR MS in order to rationalise differences in the compounds’ cytotoxicities.

**Figure 4:**
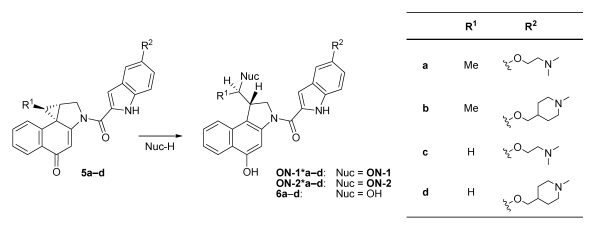
Reaction of the drugs **5a**-**d** with the DNA oligonucleotides **ON**-**1** or **ON**-**2** under formation of the alkylated oligonucleotides **ON**-1*a-**d** and **ON**-2*a-**d**, respectively, or hydrolysis of the drugs to give the alcohols **6a**-**d**.

An alkylation of the DNA oligonucleotides **ON**-**1** and **ON**-**2** by the drugs **5a**-**d** results in the form­ation of covalent adducts denoted by **ON**-1*a-**d** and **ON**-2*a-**d**, respectively (Figure 4). In the absence of suitable nucleophiles like DNA, the drugs are partially hydrolysed to give the inactive alcohols **6a**-**d**.

For the mass spectrometric investigations, the hydrochlorides of *seco*-drugs **4a**-**d** were incubated with the DNA oligo­nucleotides in water for 24 h at 25 °C. The samples were then diluted with equivalent amounts of methanol and investigated directly by means of ESI-FTICR mass spectro­metry without preliminary purification or enrichment of the products as described previously [16,18,19]. Covalent adducts were identified by comparison of calculated and found masses and alkylation positions were determined by identification of the characteristic fragment ions formed from the alkylated oligonucleotides applying capillary skimmer dissociation (CSD) [16,18,19,29,30,31,32].

If only one of the two oligonucleotides of the double-stranded DNA was alkylated, the percentage of alkylation of **ON**-**1** or **ON**-**2** was calculated based on the relative peak intensities of the corresponding isotope peaks of the unreacted oligonucleotides of **ON**-**1** and **ON**-**2** after 24 h of incubation as compared to the same ratio after 0 h of incubation. In case both oligonucleotides were alkylated, the preferred alkylation of one of the two oligo­nucleo­tides was determined based on the relative peak intensities of the covalent adducts. Analogously, if an oligonucleotide was alkylated in more than one position, the preferred binding site was determined based on the relative peak intensities of the respective characteristic fragment ions. The percentage of alkylation of the single-stranded oligo­nucleotide **ON**-**1** of ds-**1** was determined by calculating the ratio of the peak intensity of the covalent adduct as compared to the unmodified oligonucleotide. 

**Figure 5:**
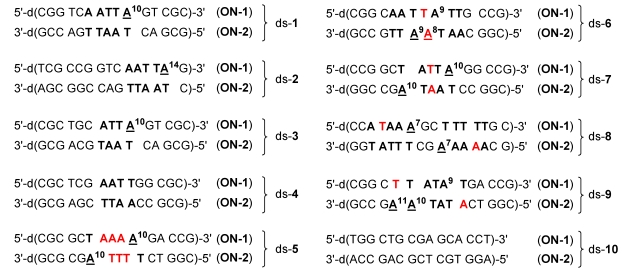
Base sequences of the ten double-stranded DNA oligonucleotides used in the present study with alkylation positions marked by underlines. Potential alkylation positions in the oligonucleotides are denoted with a superscript and differing nucleo­bases in the 5'‑direction of potential competing binding sites are marked in red.

Figure 5 and Table 2 show the results of the mass spectrometric investigations (as described above) in terms of the main alkylation site and the percentage of alkylation. In Figure 5, alkylation positions are denoted by underlines and important differences in the base sequence in the 5'‑direction of potential competing binding sites of **ON**-**1** as compared to **ON**-**2** are marked in red. Notably, all drugs alkylated mainly the nucleobase adenine (A) in AT-rich DNA regions of at least four consecutive AT base pairs with the alkylated adenine situated at the 3'-end of this sequence. This sequence selectivity could also be observed for the natural products CC‑1065 and duocarmycin SA [5] and the alkylation of the oligonucleotide **ON**-**1** of ds-**4** by the drug **5d** is the only exception hereof. Furthermore, all drugs prefer the nucleobase adenine (A) over thymine (T) in the first, second and third position in the 5'-direction of the binding site as is obvious from the preferred binding positions in the oligonucleotides ds-**6**, ds-**7** and ds-**8**, respectively (Figure 5 and Table 3). Hence, the drugs preferably alkylate the oligo­nucleotides **ON**-**2** of these double-stranded oligonucleotides instead of the oligonucleotides **ON**-**1**.

**Table 2:** Alkylation efficiency and alkylation position after incubation of the DNA oligo­nucleotides ds‑**1**-ds‑**10** and **ON**-**1** of ds-**1** with the hydrochlorides of **4a**-**d** in a 1:1 ratio for 24 h at 25 °C.

	Alkylation [%]				Alkylation position			
DNA	4a	4b	4c	4d	4a	4b	4c	4d
ds-1	75	53	46	28	A^10 ^(ON-1)			
ds-2	60	66	9	31	A^14 ^(ON-1)			
ds-3	55	67	29	32	A^10 ^(ON-1)			
ds-4	- ^a^	- ^a^	- ^a^	32	- ^b^			ON-1 ^a^
ds-5	ON-1 and ON-2 ^c^			6	A^10 ^(ON-1) ≥ A^10 ^(ON-2)			A^10 ^(ON-1)
ds-6	30	20	17	13	A^8 ^(ON-2) ≥ A^9 ^(ON-2)			
ds-7	ON-1 and ON-2 ^c^				A^10 ^(ON-2) ≥ A^10 ^(ON-1)			
ds-8	ON-1 and ON-2 ^c^				A^7 ^(ON-2) ≥ A^7 ^(ON-1)			
ds-9	77	82	21	30	A^10 ^(ON-2) ≥ A^11 ^(ON-2)			
ds-10	- ^a^	- ^a^	- ^a^	- ^a^	-^ b^			
ON-1 of ds-1	20	25	- ^a^	- ^a^	-^ b^			

^a ^No significant amount of alkylation could be detected. ^b ^The alkylation position could not be deter­mined due to the low abundance of the covalent alkylation product or of the specific fragments of the latter. ^c^ Since both oligonucleotides were alkylated, no percentage of alkylation could be determined.

In addition, all drugs show a preference for purine bases (A or G) over pyrimidine bases (C or T) in the first position in the 3'-direction of the binding site. This results for example in the alkylation of A^8^ of **ON**-**2** in ds‑**6** additionally to A^9^ of the same DNA oligonucleotide (Figure 5, Table 3).

**Table 3:** Prefered nucleobases in the vicinity of the alkylation position (0) as observed after incubation of the hydrochlorides of *seco*-drugs **4a**-**d** with the DNA oligonucleotides ds‑**1**-ds‑**10**.

	5'	4	3	2	1	0	-1	3'
4a, 4c, 4b, 4d		A/T > G/C, G/C > A/T	A > T	A > T	A > T	A	A/G > C/T	

Interestingly, the *seco*-drugs **4a** and **4c** containing a right hand dimethylamino-ethoxyindole subunit prefer AT base pairs in the fourth position in the 5'-direction of the binding site with the AT-rich sequence being located in the middle of the double strand. This is obvious due to the lower alkylation efficiencies of the double-strands ds-**2** and ds-**3** as compared to ds‑**1**. In contrast, the *seco*-drugs **4b** and **4d** containing a right hand morpholinoindole subunit prefer CG base pairs in the fourth position in the 5'-direction of the binding site and AT-rich sequences located at the end of the double strand. This can be seen by the higher alkylation efficiencies of the double-strands ds-**6** and ds-**7** in comparison to ds-**1**. Furthermore, all drugs showed a much higher reactivity against the double-stranded DNA oligo­nucleotide ds‑**1** than against the respective single-stranded oligonucleotide **ON**-**1** of ds-**1** (Table 2).

In summary, all drugs show a high selectivity for adenines in AT-rich DNA regions and differ only slightly in the preferred base sequence. Thus, differences in cytotoxicity are presumably not due to differences in sequence selectivity. Unexpectedly, also the alkylation efficiency regarding the same DNA oligonucleotide does not seem to correlate with the cytotoxicity of the drugs because, with the exception of ds-**4**, **4a** and **4b** alkylated the DNA with a significantly higher efficiency than the *seco*-drugs **4c** and **4d** even though they show a lower cytotoxicity as observed in the cell culture assays than the latter (Table 2).

### 2.2. Investigations on the Reaction Kinetics of the Seco-Drugs

Consequently other factors seem to influence the biological activity of these alkylating agents and it could be argued that differences in the rate of the cyclisation reaction of the *seco*-drugs **4a**-**d** to give the drugs **5a**-**d** or maybe differences in the rate of formation of the DNA adducts might play a crucial role. Therefore, the hydrochlorides of *seco*-drugs **4a**-**d** were incubated with the double-stranded DNA oligo­nucleo­tides in buffer or water for up to 24 h at 25 °C and the kinetics of the reactions were investigated using HPLC. Since covalent and non-covalent adducts of the DNA and the drugs elute with the same retention time under the conditions of these measurements [16], mixtures of both covalent and non-covalent adducts are denoted ds‑x^(^*^)^a-**d** in the following whereas purely covalently bound adducts as detected by means of mass spectrometry are denoted ds‑x*a-**d**. For evaluation, the area under the curve (AuC) in the HPLC chromatograms of the respective *seco*-drugs and their derivatives was determined based on their absorption of light at *λ* = 350 nm because at this wavelength the absorption of unmodified DNA can be neglected. It should be noted that the results obtained are only semi-quantitative, since we did not correct the calculated concentrations according to the molar extinction coefficients of the *seco*-drugs and their derivatives; however, it can be assumed that the extinction coefficients are approximately the same. Indeed, the results allow a good and straight­forward comparison of the reactivity of the *seco*-drugs **4a**-**d** and their drugs **5a**-**d**, respectively.

**Figure 6:**
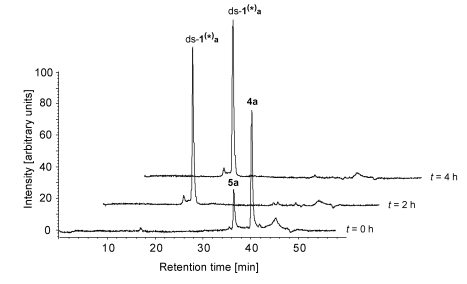
HPLC chromatograms (*λ* = 350 nm)obtained 0-4 h after starting the incubation of the hydrochloride of *seco*-drug **4a** with ds-**1** in phosphate buffer (pH 7).

Figure 6 shows a series of HPLC chromatograms obtained at 0-4 h after starting the incubation of the hydrochloride of *seco*-drug **4a** with ds-**1** in phosphate buffer (pH 7). As can clearly be seen, the *seco*-drug cyclises to give the corresponding drug **5a** very rapidly and the formation of the respective DNA-adduct ds-1^(^*^)^a has finished already after 2 h. Table 4 shows the resulting AuCs determined after up to 6 hours of incubation of a 1:1 mixture of the hydrochlorides of the *seco*-drugs **4a**-**d** with the DNA in phosphate buffer (pH 7).

The *seco*-drugs **4a** and **4b** cyclise rapidly to give the corresponding drugs **5a** and **5b** which subsequently form the respective DNA adducts ds-1^(^*^)^a and ds-1^(^*^)^b within two hours. Hence, no free *seco*-drugs or drugs can be observed after two hours of incubation. In contrast, the more cytotoxic *seco*-drugs **4c** and **4d** cyclise to the corresponding drugs **5c** and **5d** with a much lower reaction rate and, moreover, the DNA adduct formation proceeds quite slowly. Thus, after six hours of incubation, DNA adduct formation is not completed and therefore, *seco*-drugs **4c** and **4d** can still be detected in considerable amounts.

**Table 4:** AuC after indicated times of incubation of the DNA oligonucleotide ds-**1** with the the hydrochlorides of *seco*-drugs **4a**-**d** in phosphate buffer (pH 7).

		AuC [%] after the indicated time of incubation			
**Reaction mixture**	**Species**	**0 h**	**2 h**	**4 h**	**6 h**
ds**-1/4a**	**4a**	78	-	-	-
	**5a**	22	-	-	-
	ds-1^(^*^)^a	-	100	100	100
ds**-1/4b**	**4b**	89	-	-	-
	**5b**	11	-	-	-
	ds-1^(^*^)^b	-	100	100	100
ds**-1/4c**	**4c**	74	45	19	12
	**5c**	26	20	14	-
	ds-1^(^*^)^c	-	35	67	88
ds**-1/4d**	**4d**	76	51	34	19
	**5d**	24	20	17	19
	ds-1^(^*^)^d	-	29	49	62

Since *seco*-drugs such as **4a**-**d** cyclise to the corresponding drugs nearly quantitatively in less than 90 minutes in buffer without DNA [16,17], **4c** and **4d** obviously are stabilised by an interaction with the DNA. This interaction is relatively weak because it can be disrupted under conditions of chromato­graphy to give back the free *seco*-drugs **4c** and **4d** as well as unchanged DNA. In addition, the formation of stable non-covalent and covalent complexes of the respective drugs **5c** and **5d** with the DNA oligonucleotides seems to be disfavoured because the amounts of ds-1^(^*^)^c ds-1^(^*^)^d increase only slowly and after prolonged incubation times, free drugs **5c** (up to 4 h) and **5d** (up to 6 h) can still be detected. Interestingly, the products of hydrolysis **6a**-**d** are not observed, indicating that the rate of nucleophilic attack by water is reduced in the presence of DNA.

Further, we investigated whether there might be differences in *seco*-drug stabilisation or DNA adduct formation correlating with the base sequence. Using again HPLC, the interaction of the four *seco*-drugs **4a**-**d** with the DNA oligonucleotides ds-**1**-ds-**10** was analysed. Table 5 and Table 6 show the respective results obtained after 24 hours of incubation of a 1:1 mixture of DNA with the hydrochlorides of **4a**-**d** in water at 25 °C.

**Table 5:** AuC after 24 h of incubation of the DNA oligonucleotides ds-**1-**ds-**10** with the hydrochlorides of *seco*-drugs **4a** and **4b** in water (pH 7).

	ds-x/4a			ds-x/4b		
**ds-x**	**4a [%]**	**5a [%]**	ds-x^(^*^)^a [%]	**4b [%]**	**5b [%]**	ds-x^(^*^)^b[%]
ds**-1**	-	-	100	2	1	97
ds**-2**	-	2	98	9	2	89
ds**-3**	-	1	99	-	4	96
ds**-4**	-	64	36	-	62	38
ds**-5**	-	1	99	3	1	96
ds**-6**	-	27	73	-	40	60
ds**-7**	-	3	97	-	3	97
ds**-8**	-	-	100	2	1	97
ds**-9**	-	1	99	-	1	99
ds**-10**	-	63	37	-	40	60

After 24 hours of incubation, only traces (≤9%) of the *seco*-drugs **4a** and **4b** were detected. Furthermore, with the exception of ds-**4**,ds-**6** and ds-**10**, nearly all detectable amounts of the drugs **5a** and **5b** which had been formed during the time of incubation, were converted to the corresponding DNA adducts ds‑x^(^*^)^a and ds‑x^(^*^)^b, respectively. Additionally, even in those cases where only small amounts of DNA adduct were formed (ds-**4**,ds-**6** undds-**10**), no hydrolysis of the drugs **5a** and **5b** to the hydroxylated derivatives **6a** and **6b** (Figure 4) occurred in contrast to a notable hydrolysis of these drugs in the absence of DNA [16]. This indicates a weak interaction of the drugs with the DNA oligo­nucleo­tides that stabilises the drugs against hydrolysis on the one hand, but disfavours the alkylation reaction on the other hand. Consistent with this observation, the formation of covalent adducts as determined by the mass spectrometric investigations is low regarding ds-**4**,ds-**6** and ds-**10**.

**Table 6:** AuC after 24 h of incubation of the DNA oligonucleotides ds-**1-**ds-**10** with the *seco*-drugs **4c** and **4d** in water (pH 7).

	ds-x/4c				ds-x/4d			
ds-x	4c [%]	5c [%]	ds-x^(^*^)^c [%]	DNA fragments [%]	4d [%]	5d [%]	ds-x^(^*^)^d [%]	DNA fragments [%]
ds**-1**	1	-	99	-	45	3	52	-
ds**-2**	13	-	87	-	17	2	81	-
ds**-3**	8	-	92	-	19	3	78	-
ds**-4**	15	68	17	-	19	68	13	-
ds**-5**	59	-	41	-	63	3	34	-
ds**-6**	4	13	53	30	27	8	48	17
ds**-7**	3	11	73	13	7	2	84	7
ds**-8**	5	-	95	-	30	3	67	-
ds**-9**	2	-	83	15	21	3	70	6
ds**-10**	29	53	18	-	33	41	26	-

**Figure 7:**
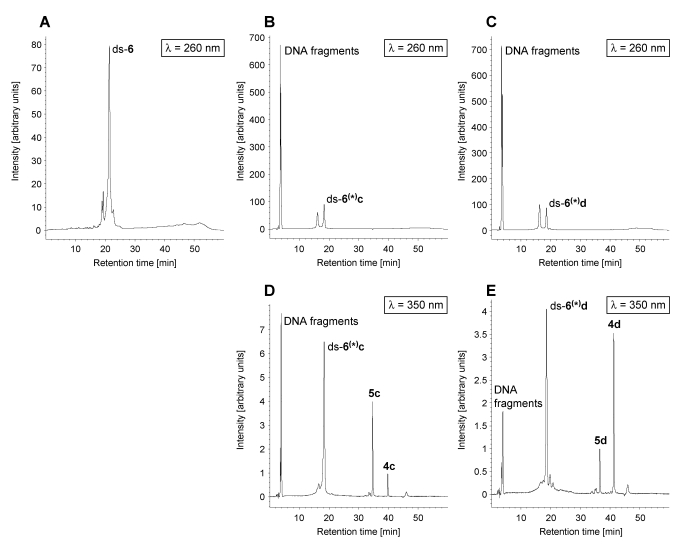
HPLC chromatograms (*λ* = 260 nm and * λ* = 350 nm) of ds-**6** in water (**Α**), a solution of DNA oligo­nucleotide ds-**6** incubated for 24 h with **4c**·HCl in water (pH 7; **B** and **D**) and a solution of DNA oligo­nucleo­tide ds-**6** incubated for 24 h with **4d**·HCl in water (pH 7; **C** and **E**).

Notably, the effect of stabilisation of the *seco*-drugs and drugs by the DNA is much more pro­nounced in the case of **4c** and **4d** as well as **5c** and **5d**, in comparison to that of **4a** and **4b** as well as **5a** and **5b**. Though in the absence of DNA all *seco*-drugs **4a**-**d** cyclise quantitatively within 90 min in buffer or cell culture media [16,17], in the presence of DNA, the *seco*-drugs **4c** and **4d** cyclise to give the corresponding drugs **5c** and **5d** to a much lower extent than their methylated analogues **4a** and **4b**. Since the formation of the drugs is the prerequisite for the alkylation reaction, this might explain why the DNA alkylation is lower in case of **4c** and **4d** as compared to **4a** and **4b**. Interestingly, the extent of drug formation depends on the kind of double-stranded DNA oligonucleotide used, indicating a specific interaction of the *seco*-drugs with the DNA. The most pronounced stabilisation of the *seco*-drugs can be observed using ds-**5**, in the presence of which only about 40% of **4c** and **4d**, respectively, are converted to the corresponding drugs in 24 hours. Nevertheless, with the exception of ds-**4**,ds-**6**, ds-**10** and partially also ds‑**7**, most of **5c** and **5d** reacted with the DNA oligonucleotides under formation of the non-covalent and covalent DNA adducts **ds**-x^(^*^)^c and **ds**-x^(^*^)^d as was already found for **5a** and **5b**. However, additional DNA fragments indicative of strand cleavage could be observed in case of ds-**6**,ds-**7** and ds-**9** after incubation with **4c** and **4d** in contrast to the respective incubations with **4a** and **4b**. Figure 7 displays HPLC chromatograms showing the DNA fragmentation exemplarily for the reaction of *seco*-drugs **4c** and **4d** with the oligonucleotide ds-**6**. DNA was detected using a wavelength of *λ* = 260 nm and the *seco*-drugs **4c** and **4d** as well as all their derivatives including DNA adducts were detected using a wavelength of *λ* = 350 nm. As can clearly be seen, the drugs **5c** and **5d** induce cleavage of the intact DNA to form DNA fragments a part of which is still covalently bound to the drug.

The high cytotoxicity of **4c** and **4d** might thus also be modulated by DNA strand cleavage. DNA lesions such as single- and double-strand breaks have been reported previously to be caused by analogues of CC-1065 and the duocarmycins such as adozelesin and bizelesin [33]. These damages can lead to cell death in case they cannot be repaired by the cellular repair machinery [34].

## 3. Experimental Section

### 3.1. Materials

The hydrochlorides of *seco*-drugs **4a**-**d** were synthesised according to previously published pro­cedures and stock solutions in DMSO were prepared [6,7,8,9]. The synthetic duplex-DNA oligomers ds-**1**-ds-**10** and the single-stranded oligonucleotide **ON**-**1** of ds-**1** were purchased from IBA (Göttingen, Ger­many) as aqueous solutions (0.1 mm) of the sodium and ammonium salts, respectively. The phosphate buffer (pH 7.0) was composed of Na_2_HPO_4_/NaH_2_PO_4_ (10 mm) and NaCl (0.1 m) in bidistilled water.

### 3.2. Incubation of **4a-d** with Synthetic DNA Oligonucleotides

Incubations of the hydrochlorides of *seco*-drugs **4a**-**d** with the double-stranded DNA oligo­nucleo­tides ds-**1** -**10** and the single-stranded DNA oligonucleotide **ON**-**1** of ds-**1** were carried out in a 1:1 ratio of *seco*-drug to DNA. For each experi­ment, an aliquot (1 µL, 5 nmol) of the stock solution of the respective *seco*-drug hydrochlorides in DMSO (5 mmol L^-1^**4a**-**d**) was mixed with an aliquot of DNA in water (50 µL, 5 nmol, 0.1 mmol L^-1^ DNA). Either, the reaction mixture was incubated at 25 °C for 24 h as such or phosphate buffer (pH 7, 50 µL) was added before starting the incubation. Samples were taken at different incubation times and analysed by means of mass spectrometry and chromatography. 

### 3.3. Electrospray Ionisation Fourier Transform Ion Cyclotron Resonance Mass Spectrometry (ESI-FTICR MS)

For mass spectrometric investigations of the reaction mixtures, samples were taken at 0 h and 24 h, diluted with an equivalent amount of methanol and the resulting solution introduced directly into the ion source of the ESI-FTICR mass spectrometer. High-resolution mass spectrometry was performed using a 7 T-FTICR-MS instrument (APEX IV, Bruker Daltonics, Billerica, USA) equipped with an APOLLO electrospray ion source and a syringe pump (74900 series, Cole-Parmer, Vernon Hills, USA) with a flow rate of 2 µL min^‑1^ for sample injection. The ions were accumulated in the hexapole region for 0.8 s and transferred subsequently into the ICR cell. For gentle desolvatisation the drying gas temperature was set to 100 °C and the capillary exit voltage to -100 V. Enhanced fragmentation of alkylated oligonucleotides was achieved by capillary-skimmer dissociation (CSD) with a capillary exit voltage of -150 V. Ions were generated in the negative ion mode.

### 3.4. High Performance Liquid Chromatography (HPLC)

HPLC separations were performed with an Agilent 1200 with DAD from Agilent Technologies, an Aquapore OD-300 Column (220 × 4.6 mm, 7 µm) from Perkin Elmer and a Bondapak^®^ C18 Column (300 × 3.9 mm, particle size 10 µm, pore size 125 Å) from Waters. Samples were eluted within 45 min with a flow rate of 1 mL min^-1^ (Aquapore OD-300) or 2 mL min^-1^ (Bondapak^®^ C18) at 28 °C by applying a two-stage gradient (0-2 min: 5% B, 2-22 min: 5→20% B, 55-45 min: 20→80% B, 45-50 min: 80% B, 50-60 min: 80→5% B). Eluent A: 0.1 mol L^-1^ triethylammonium acetate buffer (H_2_O, pH 7.0). Eluent B: 0.1 mol L^-1^ triethylammonium acetate buffer (80% acetonitrile and 20% water, pH 7.0). The absorption of the drugs and their derivatives at *λ* = 350 nm was used to calculate the AuCs.

## 4. Conclusions

In summary, compounds **4c** and **4d** which are *seco*-analogues of the natural products CC‑1065 and duocarmycin SA, show a much higher cytotoxicity in cell culture investigations using a human bronchial carcinoma cell line than the respective derivatives **4a** and **4b** containing a methyl group instead of hydrogen in the pharmacophoric unit. In order to understand the structure activity relationships underlying the differences in cyto­toxicity, the reactivity of **4a**-**d** against DNA as the proposed cellular target molecule was investigated by means of mass spectrometry and high performance liquid chromatography.

The *seco-*drugs **4a**-**d** form the respective drugs **5a**-**d** *in situ*, and the latter act as DNA alkylating agents. The drugs show high affinity to AT-rich DNA sequences and alkylate adenines at the 3'‑end of such sequences with high selectivity. All *seco*-drugs **4a**-**d** are stabilised by the interaction with double stranded DNA oligonucleotides. This stabilisation delays the cyclisation reaction that gives the respective drugs **5a**-**d**, and the effect is much more pronounced for *seco*-drugs **4c** and **4d** as compared to **4a** and **4b**. As a consequence, **4c** and **4d** are protected against hydrolytic deactivation and cyclise to the respective drugs **5c** and **5d** with a much lower reaction rate in the presence of DNA. In addition, the drugs **5c** and **5d** are less reactive than **5a** and **5b** and therefore alkylate double-stranded as well as single-stranded DNA much more slowly. The stability of the derivatives in the presence of DNA oligonucleotides correlates very well with the cytotoxicity of the compounds in cell culture investigations: The stronger the stabilisation of the *seco*-drugs and drugs by interaction with DNA oligonucleotides, the higher is their cytotoxicity. This observation is similar to results previously obtained by *Boger et al.* [35], in which a parabolic relationship between the cytotoxic potency of derivatives related to **5a**-**d** and their stability against solvolysis was demonstrated. Furthermore, **5c** and **5d** seem to cause DNA fragmentation at room temperature whereas **5a** and **5b** do not. Based on our investigations, we assume that the mechanism responsible for the very high cytotoxicity of CC‑1065, the duocarmycins and of related synthetic analogues is an over-stabilisation of the DNA double strand by non-covalent interactions, which prohibits the repair, transcription or replication of DNA and therefore induce apoptosis. In the literature, the cytotoxicity of CC-1065, the duocarmycins and related compounds is always explained by their alkylation properties. However, we think that the alkylation only serves for an irreversible fixation of the compounds in the minor groove of the DNA and the astounding base selectivity of the alkylation is only a question of proximity. Thus, the discussion that the adenine which is alkylated might be a so called hot spot explaining the very high cytotoxicity of these compounds compared to other alkylating drugs as cyclophosphamide is in our belief not meaningful anymore. 
